# MMP-9 Signaling Pathways That Engage Rho GTPases in Brain Plasticity

**DOI:** 10.3390/cells10010166

**Published:** 2021-01-15

**Authors:** Izabela Figiel, Patrycja K. Kruk, Monika Zaręba-Kozioł, Paulina Rybak, Monika Bijata, Jakub Wlodarczyk, Joanna Dzwonek

**Affiliations:** Nencki Institute of Experimental Biology, Polish Academy of Sciences, 3 Pasteur Street, 02-093 Warsaw, Poland; i.figiel@nencki.edu.pl (I.F.); p.kruk@nencki.edu.pl (P.K.K.); m.zareba-koziol@nencki.edu.pl (M.Z.-K.); p.rybak@nencki.edu.pl (P.R.); m.bijata@nencki.edu.pl (M.B.); j.wlodarczyk@nencki.edu.pl (J.W.)

**Keywords:** small Rho GTPases, extracellular matrix, MMP-9, synaptic plasticity, post-translational modifications

## Abstract

The extracellular matrix (ECM) has been identified as a critical factor affecting synaptic function. It forms a functional scaffold that provides both the structural support and the reservoir of signaling molecules necessary for communication between cellular constituents of the central nervous system (CNS). Among numerous ECM components and modifiers that play a role in the physiological and pathological synaptic plasticity, matrix metalloproteinase 9 (MMP-9) has recently emerged as a key molecule. MMP-9 may contribute to the dynamic remodeling of structural and functional plasticity by cleaving ECM components and cell adhesion molecules. Notably, MMP-9 signaling was shown to be indispensable for long-term memory formation that requires synaptic remodeling. The core regulators of the dynamic reorganization of the actin cytoskeleton and cell adhesion are the Rho family of GTPases. These proteins have been implicated in the control of a wide range of cellular processes occurring in brain physiology and pathology. Here, we discuss the contribution of Rho GTPases to MMP-9-dependent signaling pathways in the brain. We also describe how the regulation of Rho GTPases by post-translational modifications (PTMs) can influence these processes.

## 1. Introduction

Accumulating data support the importance of interactions between extracellular matrix molecules with pre- and postsynaptic neuronal elements for the formation and remodeling of central synapses. One of the key components of the extracellular matrix (ECM) that plays a role in synaptic plasticity, learning and memory formation is matrix metalloproteinase 9 (MMP-9) [1,2]. It is implicated in ECM remodeling via cleavage of ECM components such as laminin and aggrecan, and cell adhesion molecules [3,4]. Several cell adhesion proteins have been proposed as substrates of MMP-9 in neurons [5,6] but their role in the structural and functional plasticity has not yet been established. At the same time, MMP-9 signaling has been shown to contribute to long-term memory, its underlying synaptic plasticity [1,2,7,8,9,10], formation and maintenance of dendritic spines [11,12,13,14], and dendritic protrusions associated with neuropsychiatric disorders (rev. in [15]). Thus, it appears to be an ideal candidate molecule responsible for synaptic remodeling, although the key substrates and downstream mechanisms of MMP-9′s enzymatic activity in synaptic plasticity and learning remain unclear [2].

It is known, however, that dynamic changes in synaptic organization require remodeling of the actin cytoskeleton, which is regulated by Rho GTPases. They constitute a family of molecular switches that regulate signal transduction pathways by interconverting between inactive GDP-bound and active GTP-bound conformational states. They can be activated by Rho-specific guanine nucleotide exchange factors (GEFs) and inhibited by GTPase-activating proteins (GAPs). Guanine nucleotide dissociation inhibitors (GDIs) sequester the GDP-bound form of some GTPases in the cytosol and prevent them from localizing to membranes or being activated by GEFs. The action of Rho GTPases can also be regulated by PTMs [16]. Rho GTPases are activated by signals originating from various cell-surface receptors and, in the GTP-bound active conformation, they interact with a range of effector proteins, including kinases, actin regulators, and adaptor proteins, leading to changes in cell behavior [17]. Thus, Rho GTPases regulate cytoskeletal rearrangements, cell motility, cell polarity, vesicle trafficking, and the cell cycle (rev. in [18]). In neurons, GTPases of the Rho family regulate spinogenesis and synaptogenesis [16], axon guidance [19], dendritogenesis, and synaptic plasticity [20]. Increasing evidence argues for the role of PTMs of Rho GTPases in controlling the structure and function of synapses [21]. Although GTPases play an important role in regulating cell function and are often at the center of many signaling pathways, their activity, specificity, and therefore cellular response depend on upstream proteins.

**Figure 1:**
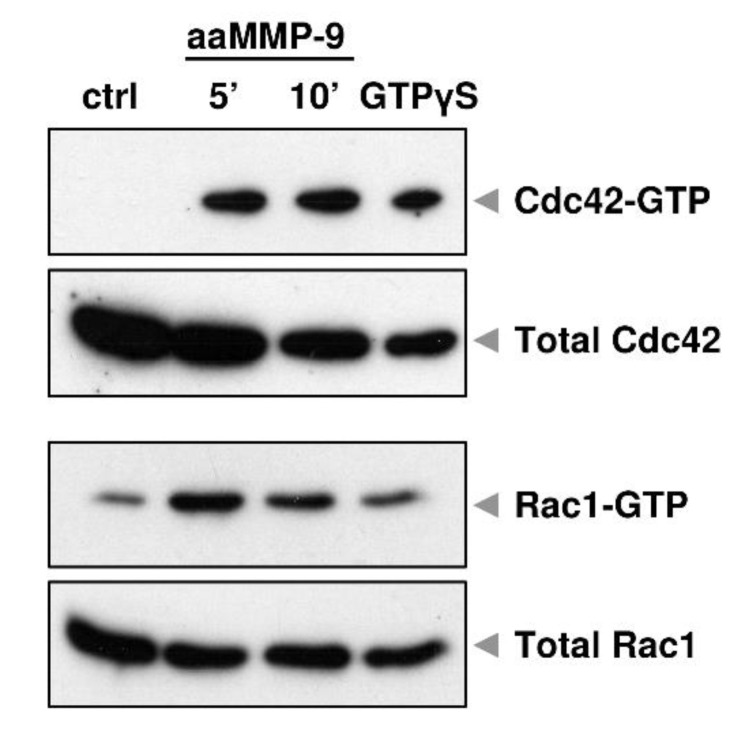
MMP-9 proteolytic activity causes Cdc42 and Rac1 activation in cultured cortical neurons. Cells were treated with autoactivaiting matrix metalloproteinase 9 (aaMMP-9) for 5 and 10 min and the activity of Rho-GTPases was analyzed using a pull-down assay. Western blots show levels of the activated form (coupled with GTP) and total levels of Cdc42 and Rac1. GTPγS served as a positive control for pull-down assay.

Our previous data indicate that the proteolytic action of MMP-9 in primary hippocampal neurons reduced the complexity of dendritic arbors, and this effect was associated with enhanced Cdc42 activity [22]. Furthermore, short-term stimulation of cortical neurons in vitro with autoactivating MMP-9 (aaMMP-9) induced rapid activation of Cdc42 and Rac1, as revealed by the pull-down assay (Figure 1). At the same time, we did not observe any changes in RhoA activity (data not shown). These results suggest that small GTPases are downstream effectors of MMP-9 signaling in the brain.

Since MMP-9 is a known modifier of the ECM [1,3,4], here we review the intracellular pathways and MMP-9 substrates that are involved in the activation of small Rho GTPases, thought to underlie ECM remodeling and synaptic plasticity. We focus on several well-described MMP-9 substrates in the central nervous system (CNS): DG (dystroglycan), CD44, ICAM-5 (intercellular adhesion molecule-5, also known as telencephalin/TLN), NLGN1 (neuroligin 1), BDNF (brain-derived neurotrophic factor), NCAM (neural cell adhesion molecule), EphB2 (ephrin type-B receptor 2), and CRMP2 (collapsin response mediator protein 2). We also discuss post-translational modifications of RhoGTPases that may play a key role in these processes.

## 2. Regulation of MMP-9 Substrates by Small Rho GTPases

### 2.1. DG

DG is a cell adhesion molecule and a central component of the dystrophin-glycoprotein complex [23]. It was originally found in skeletal muscle but is also highly expressed in other developing and adult tissues. DG is composed of two non-covalently linked α and β subunits derived from post-translational processing of a single protein encoded by the *Dag1* gene [24]. Extracellular α-DG is highly glycosylated and therefore interacts with several ECM proteins, such as laminin, agrin, perlecan, and neurexin [25,26,27]. The transmembrane β-DG anchors α-DG to the cell membrane via the N-terminal domain and interacts with the cytoskeletal proteins dystrophin and utrophin via the C-terminal cytoplasmic domain [28]. Thus, DG forms a structural linkage between ECM and the intracellular actin cytoskeleton. However, this linkage must be dynamically modulated in contexts where cellular plasticity is required, such as cell migration, wound healing, or the function of neuronal synapses. One of the mechanisms regulating the interaction between DG and ECM is post-translational cleavage by matrix metalloproteinases.

The role of DG in neurons and glia has been investigated using mice carrying a floxed *Dag1* allele because constitutive deletion of *Dag1* causes early embryonic lethality [29]. These studies indicate a crucial role for extracellular α-DG interactions since the cerebral cortex developed normally in transgenic mice that lacked the DG intracellular domain [30]. On the other hand, β-DG serves as a scaffold for various proteins involved in signal transduction pathways, such as the adapter protein Grb2 (growth factor receptor-bound protein 2), the kinases ERK (extracellular-signal-related kinase), and MAPK (mitogen-activated protein kinase) [31,32], and therefore deletion of β-DG may lead to serious disturbances in cell signaling processes.

In the adult mouse brain, DG was found at postsynaptic specializations of the cerebral cortex, hippocampus, olfactory bulb, and cerebellum [33]. Immunocytochemical studies in dissociated neuronal cultures revealed that DG is selectively associated with inhibitory GABAergic synapses and can be used as a marker of these synapses [34,35]. It has also been shown that prolonged elevation of neuronal activity leads to increased expression of glycosylated DG and the aggregation of α-DG and GABAA receptors at the postsynaptic surface [36]. Interestingly, this inhibitory upscaling is partly reproduced by the addition of agrin to the culture medium, indicating that ligand-induced signaling through DG is responsible for the modulation of GABAergic synaptic strength. In the light of these data, it is not surprising that breaking the link between DG subunits may lead to a disruption of DG interactions with other proteins, which is of great importance for physiological processes taking place in the CNS.

It has been established that β-DG is a native substrate of MMP-9 in the brain that is cleaved in response to enhanced neuronal activity [5]. In this study, stimulation of neuronal cultures with either glutamate or bicuculline leads to the formation of a 30 kDa product, readily detectable by Western blot. Therefore, the β-DG cleavage assay is commonly used to estimate the endogenous MMP-9 activity. Since then, MMP-9-dependent proteolytic cleavage of β-DG has been shown to contribute to physiological synaptic plasticity, learning and memory formation. Enhanced MMP-9 activation and subsequent β-DG proteolysis in the mouse amygdala, hippocampus, and prefrontal cortex have been linked to fear conditioning [37]. Increased cleavage of β-DG has also been found in protein lysates from primary cortical neurons exposed to a mixture of forskolin, rolipram, and picrotoxin, i.e., chemically induced long-term potentiation (cLTP) that is known to upregulate endogenous MMP-9 activity [13]. Importantly, this enhanced proteolytic activity was accompanied by the structural and functional remodeling of dendritic spines. Live imaging of hippocampal neurons revealed that cLTP stimulation led to the formation of spine head protrusions (SHPs). Notably, spines with SHPs contained more AMPA receptors, providing evidence for the involvement of MMP-9 in alterations of synaptic connectivity.

On the other hand, enhanced proteolysis of β-DG has been associated with several neuronal pathologies ranging from a stroke to neurodegeneration and epileptogenesis. The appearance of a 30 kDa β-DG degradation product has been detected in the hippocampus of mice injected intraperitoneally with PTZ (pentylenetetrazole), a proconvulsant and GABAA receptor antagonist [5,38]. Increased proteolytic degradation of β-DG has also been observed in organotypic hippocampal cultures treated with kainic acid, known to induce neurotoxicity [38]. Importantly, the application of DP-b99, a membrane-activated zinc chelator, effectively reduced β-DG cleavage and had a neuroprotective effect.

There is still little direct experimental evidence linking MMP-9-dependent cleavage of β-DG in the CNS with activation of Rho GTPases. A study on the involvement of DG in dendritic morphogenesis showed that MMP-9-mediated β-DG proteolysis inhibits dendritic tree growth and arborization of hippocampal primary neurons, and these structural changes are closely related to increased Cdc42 activation [22]. It is well established that synaptic formation, modulation, and stabilization depend on the dynamics of actin filaments, which (as mentioned above) is strictly regulated by Rho-family GTPases. Since β-DG has been shown to interact directly with a RhoGEF (Dbl) that is responsible for Cdc42 activation and thus leads to filopodia formation in fibroblasts [39], it is reasonable to speculate that DG may also play an important role in synaptic remodeling. However, further studies are required to confirm this statement.

### 2.2. CD44

CD44 is a transmembrane receptor for hyaluronan (HA), which represents the main component of the ECM influencing both structural and functional aspects of neuronal plasticity [40,41]. CD44 is a highly glycosylated protein that consists of several functional domains. While the extracellular N-terminal domain is responsible for the binding of HA and other ligands, e.g., matrix metalloproteinases, the C-terminal cytoplasmatic region is involved in the interaction with many different effectors including small Rho GTPases [42].

CD44 plays an important role in physiological and pathological processes in the nervous system. It was shown to be involved in the regulation of axon growth, dendritic tree arborization, calcium signaling, synaptogenesis and synaptic plasticity, astrocyte morphology, neurogenesis, epileptogenesis, ischemia, multiple sclerosis, Alzheimer’s disease, and brain tumors (rev. in [43]).

Proteolytic cleavage of the CD44 ectodomain by MMP-9 was initially described in glioblastoma cells [44]. Recently, we identified CD44 as an MMP-9 substrate in neurons [45]. We showed that 5-HT7 receptor stimulation increases local MMP-9 activity, triggering extracellular cleavage of CD44 by MMP-9, followed by Cdc42 activation to regulate synaptogenesis, long-term potentiation (LTP), and the structural plasticity of dendritic spines.

Moreover, CD44 modulates the functional and structural plasticity of dendritic spines via the Rho GTPases-dependent mechanism [46]. Specific knockdown of CD44 in neurons cultured in vitro resulted in changes in dendritic spine morphology, reduced number of functional synapses, and disturbed activity-dependent structural plasticity of dendritic spines. Using FRET-based biosensors of Rho GTPases, we have shown that CD44 regulates the activity of RhoA, Rac1, and Cdc42 in dendritic spines. Higher levels of activated Cdc42 and Rac1 and lower activation of RhoA were observed in CD44-depleted neurons. Defects in dendritic spine morphology that were induced by CD44 knockdown were rescued by inhibition of Cdc42 activity.

Astrocytic processes that are associated with synapses, enwrapping, and interacting with dendritic spines and synaptic terminals actively contribute to structural plasticity of dendritic spines, synapse formation, and stabilization [47,48,49,50,51,52,53,54,55]. Interestingly, CD44 regulates astrocyte morphology via Rac1 signaling [56]. Its depletion in astrocytes leads to stellate-like morphology of the cells and elevated level of Rac1 activity, whereas the overexpression results in the increased cell area, flattened morphology of astrocytes, and reduced activity of Rac1. Moreover, CD44 knockdown-induced stellation was inhibited by blocking Rac1 activity.

### 2.3. ICAM-5

The neuron-specific ICAM-5 belongs to the immunoglobulin (Ig) superfamily of adhesion proteins and is a neuronal cell surface protein. The external part of ICAM-5 is formed by 9 Ig domains, where binding sites for integrins and vitronectin are localized. The cytoplasmic domain of ICAM-5 interacts with α-actinin and ERM (ezrin, radixin, moesin) family of cytoplasmic proteins [57].

ICAM-5 shows low expression during embryonic development but increases rapidly after birth [58]. At the same time, dendritic elongation and branching as well as synapse formation, occur. ICAM-5 stimulates neurite outgrowth and plays an important role in spine morphogenesis [59,60]. Interestingly, unlike other adhesion molecules, ICAM-5 inhibits the maturation and stabilization of synapses. This adhesion molecule is present at the soma and dendrites and is particularly abundant in dendritic filopodia to support their formation and maintenance. ICAM-5 deficiency increases spine maturation, while its overexpression favors filopodia formation, thereby slowing spine morphogenesis.

Abnormally high expression levels of ICAM-5 were found in the brains of MMP-2- and MMP-9–deficient mice [61]. This suggested that the MMPs are responsible for the proteolytic processing of ICAM-5. Indeed, long-term treatment (16 h) of hippocampal cultures with N-methyl-D-aspartic acid (NMDA) or α-amino-3-hydroxy-5-methylisoxazole-propionic acid (AMPA) led to the MMP-2- and MMP-9-dependent shedding of ICAM-5 accompanied by spine enlargement. Likewise, short-term exposure (15-30 min) of primary cortical neurons to NMDA, as well as high-frequency tetanic stimulation of hippocampal slices, also promote MMPs-dependent cleavage of ICAM-5. [62]. These findings indicate that enhanced neuronal activity induces MMPs-mediated shedding of membrane-bound ICAM-5 that may facilitate the maturation of dendritic spines.

MMP-9 has been also implicated in ECM degradation, synaptic dynamics, and experience-dependent plasticity in the mouse visual cortex [63,64,65,66]. The subcellular localization of ICAM-5 in the visual cortex is developmentally regulated and depends on MMP-9 activity [67]. Ultrastructural studies of ICAM-5 distribution before and during the critical period for ocular dominance plasticity showed that during early visual development, ICAM-5 occurs mainly in immature dendritic protrusions, while in later developmental stages it is present in dendritic shafts. However, this developmental shift in ICAM-5 localization does not occur in MMP-9 knockout mice. Additionally, in the absence of MMP-9, the ICAM-5 immuno-reactive elements make significantly more contacts with axonal terminals [68]. Together this may indicate that MMP-9 cleaves ICAM-5 to determine its subcellular localization and in turn induces spine maturation during the development of the visual cortex.

Since the spines are largely devoid of ICAM-5, its exclusion from the filopodia surface appears to be required in the process of the dendritic spine maturation. Using HeLa cells and primary hippocampal neurons, it was demonstrated that internalization of ICAM-5 from the cell membrane is mediated by the small GTPase ADP-ribosylation factor 6 (ARF6), recruited by ICAM-5 and activated by GEF, EFA6A [69]. Endocytosis of this adhesion molecule affects filopodia-to-spine transition and requires Rac1-mediated dephosphorylation and release of actin-binding ERM proteins from ICAM-5 [70]. In summary, ICAM-5 is cleaved by MMPs upon activation of glutamate receptors or degraded by RhoGTPase-dependent endocytosis, and both processes promote the maturation of dendritic spines.

### 2.4. NLGN1

NLGN1 is a transmembrane cell adhesion protein that belongs to the highly conserved neuroligin family. Five isoforms of NLGN have been identified in mammals, each exhibiting a specific pattern of expression and subcellular distribution [71,72]. NLGN1 is located mainly on the postsynaptic side of excitatory synapses [73,74]. The extracellular domain of NLGN1 interacts with presynaptic neurexin1β (NRX1β), forming a trans-synaptic complex [75,76], whereas its intracellular domain binds to postsynaptic density protein-95 (PSD-95; [77]). PSD-95 is required for efficient NLGN1 surface expression in cultured neurons and importantly, PKA-dependent phosphorylation of NLGN1 controls its interaction with PSD-95 [78].

Many studies indicate that NLGN1 plays a crucial role in synapse maturation and brain function [72,79,80,81]. Live imaging of primary cortical neurons from NLGN1-deficient mice has shown destabilization of excitatory synaptic organization and impaired tenacity of these synapses [82]. NLGN1-deficient mice also exhibit impaired LTP [83,84], and NLGN1 knockdown in epileptic rats reduced seizure severity. Whole-cell patch-clamp recordings of pyramidal hippocampal neurons from epileptic rats revealed a decrease in the amplitude of NMDA receptor-dependent excitatory postsynaptic currents [85]. Moreover, silencing of NLGN1 in mice blocks the storage of fear memory by reducing NMDAR-mediated currents [83]. Importantly, a genomic microarray test detected partial loss of NLGN1 in a patient with seizures and severe intellectual disability [86]. Mutations in the NLGN1 gene have been proposed to be associated with autism and other neuropsychiatric disorders [87,88].

Neuronal activity-driven cleavage of NLGN1 by MMP-9 was reported by Peixoto et al. [10]. Notably, the authors showed that MMP-9-dependent shedding of NLGN1 leads to the destabilization of presynaptic NRX1β and downregulation of synaptic transmission. Recent data suggest that the soluble extracellular domain of NLGN1 binds to metabotropic glutamate receptor 2 (mGluR2), and thereby decreases synaptic strength [89].

There have been several reports describing mechanisms by which NLGN1 affects actin reorganization and spine stability. An elegant study by Liu et al. demonstrated that the cytoplasmic C-terminal domain of NLGN1, remaining after proteolytic cleavage, may interact with the spine-associated Rap GTPase-activating protein (SPAR) leading to activation of Rap1/Rac1/LIMK1/cofilin pathway and subsequent spine growth and synapse development [90]. Furthermore, NLGN1 appears to promote excitatory synaptogenesis by cooperation with the postsynaptic adhesion-G protein-coupled receptor (A-GPCR) BAI1, which also results in local activation of Rac1 within spines [91]. Recently, quantitative proteomic analysis of rat brain lysate revealed that NLGN1 interacts with Kalirin-7, an essential RhoGEF of the postsynaptic density. NLGN1-mediated spine formation and glutamatergic synapse function require RhoGEF signaling, indicating that Kalirin-7 is a crucial intracellular effector of NLGN1 function [92].

### 2.5. BDNF

BDNF is a pleiotropic protein, a member of the neurotrophic factor family, playing an important role in neuronal growth and differentiation [93,94,95]. BDNF exerts its biological function mainly upon binding to tyrosine kinase B (TrkB) receptors [96,97]. The existence of multiple alternative splicing variants of BDNF [98,99,100] and the regulation of its expression by several promoters [101,102] make this neurotrophic factor a variable regulator orchestrating neurite development, learning, and memory formation in the young and adult brain. BDNF is expressed in neuronal and non-neuronal cells, and its tissue-specific localization is developmentally regulated [103]. Neuronal BDNF immunoreactivity was found in several regions of the CNS, as well as in the peripheral and enteric nervous system [104,105]. However, BDNF occurs prominently in the brain and can be found in the hippocampus, cortex, amygdala, striatum, and hypothalamus (rev. in [106]), and localized mostly at presynaptic terminals [107,108] and postsynapses [109,110]. In addition to neurons, astrocytes and microglia are also important sources of BDNF [111,112]. Altered levels of BDNF in the CNS are involved in the pathogenesis of neurodegenerative diseases (e.g., Alzheimer’s and Parkinson’s) [113] and mental disorders (e.g., depression and schizophrenia) [114]. Abnormalities in the regulation of BDNF secretion also affect the proper development of neonates and probably contribute to autism spectrum disorders [115,116].

BDNF is synthesized as a precursor proBDNF, about 32 kDa protein [117], that is responsible for correct folding of the mature form (mBDNF). Importantly, proBDNF can target the secretory pathway and through interaction with the p75 neurotrophin receptor (p75^NTR^) may act as an active signaling molecule. The conversion of proBDNF to mBDNF occurs through many different proteolytic mechanisms, leading to the release of a polypeptide of approximately 14 kDa. ProBDNF can be cleaved intracellularly by serine proteases, such as convertases PC1/3 and PC7 [118,119], and/or furin [120]. However, at synapses, this neurotrophic factor is predominantly secreted as proBDNF and then digested by extracellular proteases. Extracellular conversion of proBDNF to mBDNF is shown to be essential for hippocampal late-phase LTP (L-LTP), and this is mediated by various proteases including tPA (tissue plasminogen activator)/plasmin [121], and/or metalloproteinases MMP-3, MMP-7, and MMP-9 [120]. Importantly, proBDNF and mBDNF can have opposing effects on neuronal structure and synaptic plasticity, and therefore the extracellular levels of both proteins are tightly controlled by neuronal activity [122,123,124,125,126,127,128].

In the PTZ kindling mouse model of epilepsy, enhanced hippocampal expression of MMP-9 was found to be associated with increased levels of mBDNF, while downregulation of this neurotrophic factor was observed in kindled MMP-9-deficient mice. In addition, decreasing mBDNF by injecting the mice with the BDNF scavenger TrkB-Fc, significantly suppressed the kindling development in wild-type mice, but not in MMP-deficient mice [129].

A growing body of evidence indicates that GTPases are potent mediators of BDNF signaling implicated in synapse formation and plasticity. The conversion of nonfunctional synaptic contacts between hippocampal neurons in vitro into functional synapses occurs through activation of the BDNF/TrkB signaling pathway and can be abolished by the transfection with a plasmid encoding dominant-negative Cdc42. Moreover, treatment of the cultures with Cdc42 activator, bradykinin, also promotes presynaptic maturation. Thus, rapid induction of functional synapses requires the activation of Cdc42, a downstream effector of TrkB [130]. In neonatal sympathetic neurons that do not express the TrkB receptor, binding of BDNF to p75^NTR^ activates the RhoA/ROCK pathway, promoting axonal degeneration [131]. This mechanism may be of general importance as p75^NTR^ is highly upregulated in most post-traumatic CNS neurons [132,133].

Postsynaptic BDNF/TrkB signaling pathway and downstream activation of small GTPases are crucial for structural and functional plasticity. In CA1 hippocampal neurons, glutamate uncaging evokes BDNF release from stimulated dendritic spines and subsequent TrkB activation on these same spines, assessed using a FRET-based biosensor for TrkB [110]. The expression of structural LTP (sLTP) in stimulated spines is associated with the distinct spatial activation of Cdc42 and RhoA [134]. Additionally, imaging the activity of GTPases in organotypic hippocampal slices from BDNF or TrkB conditional knockout mice revealed the spatiotemporal activation patterns of these GTPases during sLTP. Notably, activation of Cdc42 is preferentially enriched in stimulated spines and overlaps the TrkB signaling pattern, while Rac1 signaling seems to spread wider than local activation, affecting nearby spines. Moreover, inhibition of Rac1 by either pharmacological treatment, or single-cell knockout, blocks sLTP in the neighboring spines, but do not affect sLTP of the stimulated spine. Finally, activation of both Cdc42 and Rac1 depends on postsynaptic, autocrine BDNF and corresponds to the local sLTP over spine-specific (Cdc42) and heterosynaptic (Rac1) domains. Interestingly, the spreading of RhoA activation is independent on BDNF/TrkB but is also necessary for synaptic crosstalk [135].

### 2.6. NCAM

NCAM is a glycoprotein from the Ig immunoglobulin superfamily with three main MW-named alternative splicing variants: NCAM-180 and NCAM-140 expressed in neurons, and NCAM-120 identified in glia. They share an identical extracellular domain, differing on membrane anchoring and cytoplasmic region. The extracellular domain of NCAM consists of 5 Ig-like domains (Ig1-Ig5) followed by 2 fibronectin III domains [136,137] and is polysialylated in the Ig5 domain. This polysialic acid modification is necessary for synaptic plasticity, formation, and consolidation of memory [138,139,140,141].

NCAM-140 is involved in the maturation of olfactory granule cell precursors [142], whereas NCAM-180 is found in postsynaptic densities of mature neurons [143,144]. NCAM plays a role in axonal and dendritic growth, synaptogenesis, and plasticity. It was shown to be required for the development and stabilization of LTP [145].

NCAM is associated with neurodegenerative and psychiatric disorders in humans such as Alzheimer’s disease, bipolar disorder, schizophrenia, and cerebral ischemic stress [146,147,148]. Elevated levels of its 105–115 kDa isoform, possibly the N-terminal cleavage product of NCAM-180, were found in the hippocampus, prefrontal cortex, and cerebrospinal fluid of patients with schizophrenia [149].

All three main isoforms of NCAM undergo MMP-dependent shedding. In cultured cortical neurons exposed to oxidative stress upregulated MMP-9 induces cleavage of NCAM-180 producing extracellular (110–115 kDa) and intracellular (65–70 kDa) ectodomains (EC-NCAM) [150]. The involvement of MMP-9-mediated proteolysis of NCAM-180 in the development of neuronal ischemic damage in vivo was confirmed in middle cerebral artery occlusion (MCAO) model mice [151]. In this study, downregulation of MMPs activity using a broad-spectrum inhibitor (GM6001) or a knockdown of MMP-9 gene expression increases the levels of NCAM-180 accompanied by a decrease in EC-NCAM. MMP-dependent shedding of NCAM occurs in primary hippocampal neurons and this effect is enhanced by ATP added to the culture medium [152]. Application of the MMP inhibitor evokes neuronal aggregation, indicating that MMP-mediated proteolytic cleavage of NCAM is involved in the regulation of neurite outgrowth. Soluble NCAM fragments were detected in the dentate gyrus of rats after the induction of LTP in vivo [153], as well as in rat hippocampal cell culture after exposure to 17β-estradiol, which mediates enhancement of LTP in vitro [154].

Ligand affinity chromatography and subsequent peptide mass fingerprinting of rat brain extracts enabled identifying several intracellular NCAM180-interacting proteins such as α- and β-tubulin, α-actinin-1, and RhoA-binding kinase-α [155]. Moreover, NCAM associates with the receptor tyrosine kinase EphA3 in murine cortical GABAergic interneurons, promoting ephrin-A5-dependent receptor clustering. This triggers EphA3 kinase signaling and downstream RhoA-GTPase activation, leading to growth cone collapse of interneurons and hence regulation of the perisomatic synapse density [156].

### 2.7. EphB2

EphB2 transmembrane receptor is a member of the largest subfamily of receptor tyrosine kinases (RTKs). It consists of a highly conserved extracellular domain necessary for ligand recognition and binding, followed by a cysteine-rich region, two fibronectin repeats, an intracellular highly conserve kinase domain that catalyzes tyrosine phosphorylation of protein substrates, a C-terminal sterile alpha motif (SAM), and a PDZ-binding motif [157].

EphB receptors bind to their corresponding ephrin-B membrane-bound ligands, thus requiring a direct cell-cell contact. Their interaction upon the activation can be bi-directional—into Eph-expressing cells (forward signaling) or ephrin-expressing cells (reverse signaling) [158]. In the nervous system, the EphB2 receptor interacts directly with NMDA receptors and its absence may cause an impairment in the long-term potentiation and depression [159]. Additionally, EphB2 plays a crucial role in axon guidance and growth cone migration [160].

It has been demonstrated that MMP-9 protein can cleave the EphB2 receptor. MMP-induced cleavage causes a prolonged EphB2 activation by sustaining its phosphorylation. That leads to strong cytoskeletal responses, recruitment of focal adhesion kinase (FAK), and RhoA activation. It is suggested that those changes may also further impact other small Rho GTPases’ signaling pathways [161]. Studies performed on the hippocampal primary cultures have shown that ephrin-B1-induced activation of the EphB2 receptor can increase RhoA activity as a result of the FAK stimulation. That suggests the shortening of dendritic filopodia and their transformation into spine-like protrusions is mediated by EphB2 [162]. The EphB2/FAK downstream signaling through RhoA/ROCK/LIMK-1 pathway partly controls the stability of mature dendritic spines by inhibiting their cofilin-mediated remodeling [163]. Furthermore, EphB2/ephrin reverse signaling through the RhoA/ROCK pathway can negatively regulate branching and axonal outgrowth [158].

One of the exceptional characteristics of the Eph/ephrin signaling is the ability to form higher-order receptor-ligand clusters that, in comparison to monomers or dimers, invoke stronger cell repulsion response through their trans-endocytosis [164,165]. This type of reverse endocytosis is highly important for axon withdrawal during growth cone collapse [166]. In vitro studies on the primary mouse cortical neurons indicate that EphB2 trans-endocytosis is positively regulated by the activity of Rac GTPase and its specific GEF—Tiam2 [167]. The downstream signaling pathway, after the EphB2/ephrin-B clustering, can also induce the synaptic concentration of the Rho-GEF Kalirin, resulting in the local activation of Rac1 and spine morphogenesis [168].

Although most studies emphasize the importance of RhoGTPases in the regulation of actin cytoskeleton, it has been confirmed that Arg kinase activated by the EphB2 receptor can trigger actin polymerization in the dendritic spine independently of RhoGTPases. It suggests that Rac1 and Cdc42 are required particularly in the reorganization of the synapse structure but not always of the dendritic region [169]. Still, the interaction of EphB2 and Zizimin1 (Cdc42-GEF), and its downstream signaling through p21-activated kinase-3 (PAK3) was shown to regulate cell surface targeting of AMPA receptors, therefore impacting synaptic plasticity [170]. Moreover, the synergistic effect of EphB2 and neural Wiskott-Aldrich syndrome protein (WASP) highly upregulates Cdc42-GEF intersectin and, by Cdc42 activation, triggers actin polymerization and spine morphogenesis [171].

EphB2/ephrin signaling pathway also includes another small GTPase—Ras, that is considered to be engaged in the long-lasting synaptic changes, learning and memory formation, and also promotes protective mechanisms in the nervous system [172,173]. It has been shown that the activation of EphB2 in the NG108 neuronal cell line leads to a reduction of the GTP-bound Ras level in the Ras/MAPK pathway, a retraction of filopodial extensions, and a collapse of the neurite structures [160,174]. Ras/MAPK signaling was also observed to be inhibited by ephrin-B3 (EphB2 ligand) through direct binding to ERK1/2, suggesting the role of EphB2/ephrin-B3 interaction in the formation of dendritic spines and the control of synapse density [175]. Apart from that, Eph2B receptor signaling may also inactivate R-Ras by the joint effects of tyrosine phosphorylation and heightened GTP hydrolysis, causing growth cone collapse in hippocampal neurons [176].

### 2.8. CRMP2

CRMP2 is a cytosolic phosphoprotein whose monomeric structure consists of a triosephosphate isomerase (TIM) barrel, a small β-sheet domain formed by the N-terminal residues, C-terminus close segments, and a C-terminal helix that is the only region on the CRMP2 surface with a positive charge potential [177]. In the brain, it is expressed in the oligodendrocytes and developing neurons, showing its abundance especially in the hippocampus, cerebellum, and olfactory system [178,179].

CRMP2 plays a crucial role in the regulation of several signaling pathways impacting axon formation, as well as growth cone guidance and collapse, thereby participating in the establishment and maintenance of neuronal polarity [180,181,182]. Its N-terminal globular domain, particularly the helix H19, binds to the soluble GTP-tubulin dimers to promote the growth of GTP-state microtubules; while the C-terminal flexible tail further binds to those polymerized microtubules to stabilize them [183].

With the use of the two-dimensional fluorescence difference gel electrophoresis (2D-DIGE) technique and tandem mass spectrometry, CRMP2 was proposed as one of the MMP-9 extracellular targets in the synapse [6]. Results derived from Western blot analysis confirmed that MMP-9 can cleave CRMP protein. Those changes may then impact the latter’s binding ability to tubulin, thereby affecting axonal elongation.

Studies on numerous Rho GTPase effectors indicate that CRMP2 is directly dependent on the activity of those small G proteins. Activated Rho/Rho-kinase signal induces phosphorylation of CRMP2 at threonine 555, hence negatively regulating its ability to bind tubulin heterodimers, which may lead to a decrease in neurite length [184]. Although most studies portray CRMP2 as a Rho substrate, it has been suggested that it can have a negative effect on both upstream and downstream of Rho signaling. In combination with active Rho or Rac GTPases, CRMP2 may act as a cyclical switch reversing their usual morphological effects, therefore stimulating dynamic neuronal shape changes [185]. Additionally, Ras protein also can play an essential role in the regulation of neuronal polarity by inhibiting phosphorylation of CRMP2 in the PI3-kinase/Akt/GSK3α/CRMP2 signaling pathway [186]. Most probably, the erroneous signaling in the Rho/ROCK/CRMP2 pathway could have lineal implications in the development of conditions like cancer, Alzheimer’s disease, Guillain Barrè Syndrome, ischemic stroke, or peripheral nerve and spinal cord injuries [187,188,189,190,191,192].

## 3. Post-Translational Modifications of Rho GTPases Involved in Neuronal Plasticity

Rho GTPases can be modified by a variety of post-translational modifications that cause differences in localization, activity, and directly affect their function [16,193]. PTMs known to regulate the role of Rho GTPases in synaptic plasticity include lipidation, phosphorylation, and ubiquitination [16,193,194].

Lipidations such as prenylation or palmitoylation are the first and most frequently described PTMs of small GTPases. This modification consists of covalent binding of a lipid group to a peptide chain, which may affect the activity of the protein and alter its subcellular localization. The location of Rho GTPases plays a critical role in determining their spatially unique functions.

Many Rho proteins cycle from the cytosol to various cellular membranes, guided by a prenylation [195,196]. Prenylation of small GTPases directs them to a plasma membrane (PM) where they are activated by interaction with GEFs. In the absence of prenyl modification, proteins remain in the cytosol and are unable to function properly [197,198]. Prenylation plays a critical role in regulating Rac1 activity in neurons by targeting Rac1 to the neuronal PM [199]. Moreover, newly synthesized Rho GTPases such as Rac1, Cdc42, or RhoA were shown to be rapidly prenylated in the cytosol and then transferred to the endoplasmic reticulum (ER) for further modifications [195,200,201,202]. Once attached to the membrane, specific GTPases carry out a variety of functions, from neurite outgrowth and guidance (Rac1) to inhibiting neuronal extensions and promoting growth cone collapse (RhoA) [203,204,205,206,207,208,209,210]. Importantly, transport of these proteins from the endoplasmic reticulum to the PM requires another modification which is palmitoylation.

Palmitoylation is a post-translational covalent binding of the palmitate to the cysteine residue of proteins by thioester bond. In contrast to prenylation, palmitoylation is a dynamic and reversible cysteine modification [211]. A recent study showed that Rac1 undergoes palmitoylation, which enhances its recruitment to the PM and induces translocation to lipid rafts [212]. This PTM targets Rac1 for stabilization at actin cytoskeleton-linked ordered membrane regions. Inhibition of Rac1 palmitoylation significantly reduces the PM localization and GTP loading of Rac1, leading to downregulation of its effector protein, p21-activated kinase (PAK). Palmitoylation might also be a regulatory mechanism for other small GTPases such as Cdc42 [213,214]. Interestingly, the canonical Cdc42 form associates with membranes through prenylation whereas the brain-specific form of Cdc42 is palmitoylated [213,214]. Although prenylated and palmitoylated isoforms coexist in the developing neurons, only palmitoylated Cdc42 is critically involved in the extension of dendritic filopodia, which develop into dendritic spines [214,215]. Importantly, in response to glutamate, Cdc42 undergoes rapid depalmitoylation and dislocates from dendritic spines. Therefore, the level of Cdc42 in dendritic spines is rapidly modified by neuronal activity and may be responsible for dynamic changes in spine morphology [214,216]. It is also worth to mention, that there are interesting differences in palmitoylation between Ras isoforms. H-Ras is stabilized by two cysteines that can bind palmitate, while N-Ras and K-Ras4A have only one modified cysteine residue [217,218,219,220,221]. On the other hand, K-Ras4B lacks a palmitoylation site [222]. The level of Ras palmitoylation could vary between membrane compartments and might even depend on the activation status of the Ras isoforms [220]. A recent study showed that activation of H-Ras and N-Ras leads to depalmitoylation and redistribution to the ER/Golgi, where they can be repalmitoylated [217,219]. This cycle is crucial for preventing the nonspecific accumulation of Ras isoforms on all membranes. Furthermore, a detailed analysis of H-Ras trafficking indicated that specific palmitoylated cysteines play different roles in the protein localization. Palmitoylation of the first cysteine stabilizes the localization of H-Ras in the PM, whereas modification of the second cysteine facilitates redistribution between cell membranes [219,223]. The different distribution of N-Ras and H-Ras proteins in the membrane compartments is crucial for the ability of the Ras proteins to control so many distinct cellular processes.

It is not surprising that Rho GTPases can also undergo phosphorylation. Several kinases are involved in this process, namely protein kinase A (PKA), protein kinase C (PKC), and Src family kinases [224]. Phosphorylation of RhoA and Cdc42 significantly increases their interaction with RhoGDI and thus plays a key role in the activation-inactivation cycle of these GTPases, controlling the actin cytoskeletal organization in the brain under physiological conditions [225]. Rac1 phosphorylation is carried out by Src and FAK [199]. Recent data suggest that Rac1 phosphorylation negatively regulates cell spreading, focal adhesion localization, and interaction with GTP, RhoGDI, GEFs, and an effector protein PAK [226]. It was also demonstrated that PKC regulates the activation of Rac1 during sLTP of dendritic spines without affecting Cdc42 [227]. Additionally, PKC-mediated phosphorylation of K-Ras4B on serine residue initiates its dissociation from the PM [228].

It is known that Rho-family GTPases can be modified by ubiquitination, which affects their cellular localization and activity state [229]. Recent studies have shown that PM-tethered H-Ras and N-Ras undergo ubiquitination, regardless of their activation state, and such modification leads to their internalization into endosomes [230]. Ubiquitination of K-Ras enhances GDP to GTP conversion and downstream signaling. Importantly, ubiquitin-mediated degradation of RhoA protein promotes neurite outgrowth [231]. Although a growing body of evidence underlines the importance of PTMs for the signaling roles of GTPases, the consequences of these modifications for the regulation of individual proteins have not yet been fully elucidated.

## 4. Conclusions

**Figure 2:**
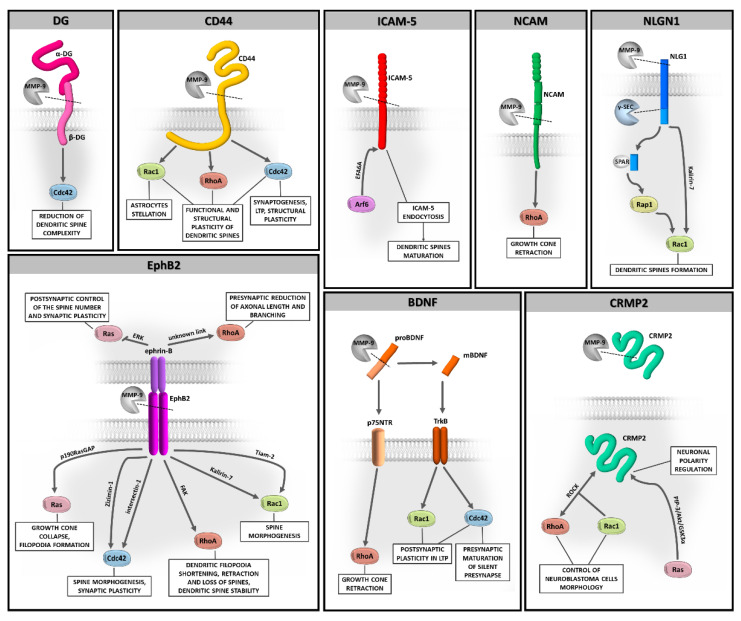
The potential relationship between MMP-9-driven substrates shedding and Rho GTPases signaling in brain plasticity.

Evidence has been accumulated that MMP-9-dependent proteolysis that occurs at the synapse is crucial for neuronal function and remodeling of synapses during physiological and aberrant plasticity. However, questions remain about the downstream cellular signaling pathways involved in these processes. In this review, we highlight the Rho family of GTPases as potentially important players in MMP-9-controlled signaling in the brain (Figure 2). We also discuss the role of PTMs of Rho GTPases, as such modifications have a direct impact on the localization and function of these proteins.

While the proteolytic cleavage of the described MMP-9 substrates and its possible regulation by activated Rho GTPases have been studied, the relationship between these two processes remains speculative. So far, only the MMP-9/CD44/Cdc42 pathway has been well proven [45]. However, the continuous development of the live-cell single-molecule tracking methods should provide an understanding of the role of Rho GTPases in the MMP-9 downstream signaling at the synapse.

## Abbreviations

AMPA	α-amino-3-hydroxy-5-methylisoxazole-propionic acid
BDNF	brain-derived neurotrophic factor
cLTP	chemically induced long-term potentiation
CNS	central nervous system
CRMP2	collapsin response mediator protein 2
DG	dystroglycan
ECM	extracellular matrix
EphB2	ephrin type-B receptor 2
ER	endoplasmic reticulum
ERK	extracellular-signal-regulated kinase
ERM	ezrin, radixin, moesin
FAK	focal adhesion kinase
GABAA	γ-aminobutyric acid type A
GAP	GTPase activating protein
GDI	guanine nucleotide dissociation inhibitor
GEF	guanine nucleotide exchange factor
ICAM-5	intracellular adhesion molecule-5
L-LTP	late-phase long-term potentiation
LTP	long-term potentiation
MAPK	mitogen-activated protein kinase
MMP	matrix metalloproteinase
NCAM	neural cell adhesion molecule
NLGN1	neuroligin 1
NMDA	N-methyl-D-aspartic acid
PAK	p21-activated kinase
PKA	protein kinase A
PM	plasma membrane
PTM	post-translational modifications
PTZ	pentylenetetrazole
ROCK	Rho-associated protein kinase
sLTP	structural long-term potentiation
TrkB	tyrosine kinase B
